# Mesenchymal Stem Cell-Educated Macrophages Ameliorate LPS-Induced Systemic Response

**DOI:** 10.1155/2016/3735452

**Published:** 2016-07-27

**Authors:** Yaoqin Hu, Chaojin Qin, Guoping Zheng, Dengming Lai, Huikang Tao, Yan Zhang, Guanguan Qiu, Menghua Ge, Lanfang Huang, Lina Chen, Baoli Cheng, Qiang Shu, Jianguo Xu

**Affiliations:** ^1^The Children's Hospital of Zhejiang University School of Medicine, Hangzhou, Zhejiang 310052, China; ^2^Shaoxing Second Hospital, Shaoxing, Zhejiang 312000, China; ^3^The First Affiliated Hospital of Zhejiang University School of Medicine, Hangzhou, Zhejiang 310003, China

## Abstract

Both bone marrow and adipose-derived mesenchymal stem cells (ASCs) have immunomodulatory effects. The goal of this study was to determine whether ASCs-educated macrophages could directly ameliorate LPS-induced systemic response in a mouse model. Mouse peritoneal macrophages were cocultured with ASCs in a Transwell system for 2 days to educate macrophages. Mice were divided into 5 groups: control, LPS, LPS + ASCs, LPS + untreated macrophages, and LPS + educated macrophages. Educated macrophages decreased lung inflammation, weight loss, pulmonary edema, and inflammatory cytokine response. In vitro, ASCs increased expression of M2 macrophages independent of direct cell-to-cell contact when macrophages were treated with LPS or serum from patients with acute respiratory distress syndrome (ARDS). When macrophages were cultured with serum from ARDS patients who were treated with ASCs or placebo in our previous clinical trial, there was no difference in M2 macrophage levels before and after ASCs treatment indicating a suboptimal response to the treatment protocol. ASCs also reduced the levels of LPS-induced proinflammatory cytokines in vitro which were mimicked by IL-10 and blocked by antibodies for IL-10 and IL-10 receptor supporting the notion that educated macrophages exert their anti-inflammatory effects via IL-10-dependent mechanisms.

## 1. Introduction

Many studies, including publications from our group [1, 2], have demonstrated compelling evidence of the benefits of mesenchymal stem cells (MSCs) from both bone marrow (BM) [3–5] and adipose tissue (adipose-derived mesenchymal stem cells, ASCs) [6–8] in animal models for lung injury and systemic inflammation. The benefit of MSCs appears to derive from the release of paracrine soluble factors such as keratinocyte growth factor [9], angiopoietin-1 [3], and prostaglandin E_2_ [10]. These factors can reduce inflammation, stabilize the injured alveolar epithelium and lung endothelium, increase the absorption of pulmonary edema fluid, and possess antimicrobial activity [11]. BM-MSCs have also been reported to attenuate sepsis via regulating host macrophages [10]. Recently, BM-MSCs were documented to shuttle mitochondria and microRNAs to macrophages, enhance macrophage bioenergetics, and inhibit macrophage Toll-like receptor (TLR) signaling [12].

Alveolar macrophages are tissue-resident or recruited cells. They form the first line of defense against airborne particles and microbes and use a variety of pattern recognition and scavenger receptors to sense and phagocytize pathogens. Upon activation, they release early response cytokines such as interferon-*γ* (IFN-*γ*), tumor necrosis factor-*α* (TNF-*α*), and interleukin-1*β* (IL-1*β*) in an interferon regulatory factor (IRF) or nuclear factor-kappa B- (NF-*κ*B-) dependent way. These cytokines stimulate neighboring alveolar epithelial cells and macrophages in an autocrine and paracrine manner to produce a variety of chemokines which in turn mediate the recruitment of neutrophils. Later on, macrophages and lymphocytes are attracted to the site of infection and facilitate the clearance of pathogens [13]. Macrophages are divided into two general categories: classical activation M1 and alternative activation M2. Classical M1 activation is driven by IFN-*γ* and TLR ligands and is an aggressive state in which macrophages attack and engulf bacteria or bacterially infected cells. M1 macrophages secrete cytokines that promote inflammation, such as IFN-*γ*, IL-6, and TNF-*α*. Alternative M2 activation describes the behavior of macrophages reacting to parasitic infection, but it is also associated with wound healing and lung fibrosis, and results in the production of anti-inflammatory cytokines such as IL-10. The M1 and M2 classifications are useful in describing proinflammatory (M1) and anti-inflammatory (M2) states [14].

The concept for “MSCs-educated macrophages” was first proposed by Kim et al. [15, 16]. They first cultured human monocytes from peripheral blood without any added cytokines to generate macrophages and then cocultured macrophages for 3 more days with culture-expanded BM-MSCs. Macrophages cocultured with MSCs consistently showed high-level expression of CD206, a marker of alternatively activated macrophages. Furthermore, these macrophages expressed high levels of IL-10 and low levels of IL-12, as determined by intracellular staining, typical of alternatively activated macrophages [15]. Anderson et al. reported that systemic infusion of macrophages pretreated with ASCs-conditioned medium inhibited colitis and polymicrobial sepsis induced by cecal ligation and puncture [17]. In another study, educated macrophages ameliorated rhabdomyolysis-induced acute kidney injury [18]. The present study was designed to test the hypothesis that educated macrophages mimic the effects of ASCs in reducing systemic response induced by LPS.

## 2. Materials and Methods

### 2.1. Human Samples

Human protocol was approved by the Research Ethics Committee at Shaoxing Second Hospital. Pooled ARDS serum samples were obtained from 3 patients treated at the ICU unit of Shaoxing Second Hospital. All subjects developed ARDS secondary to bacterial pneumonia. Subjects were mechanically ventilated for 2–4 days. The severity of ARDS was classified as moderate to severe based on the Berlin definition [19]. Serum samples from ARDS patients treated with placebo and ASCs were obtained from a clinical trial conducted by our group [20].

### 2.2. Mice

C57BL/6 mice (8–12 weeks old; Shanghai Laboratory Animal Center, Shanghai, China) were used for the study. All mice were housed in the Zhejiang University Laboratory Animal Center. Animal experiment protocols were approved by the review committee from Zhejiang University School of Medicine and were in compliance with institutional guidelines.

### 2.3. Cell Culture

For macrophages, C57BL/6 mice were injected intraperitoneally with 2 mL of 3% thioglycollate. After 3 days, the peritoneal macrophages were collected by lavage, characterized, and counted using a Beckman Coulter counter. Macrophages were maintained in medium containing Dulbecco's Modified Eagle's Medium- (DMEM-) low glucose supplemented with 1% penicillin and streptomycin and 8% fetal bovine serum (FBS) (Life Technologies, Grand Island, NY) plus EGF and FGF (R&D Systems, Minneapolis, MN) at 37°C in a humidified incubator containing 5% CO_2_. Normal human ASCs were purchased from ATCC (Cat. number PCS-500-011, passage 2, Manassas, VA). Cells were cultured in the same culture medium above at a density of 4000cells/cm^2^. Cultures were maintained at 37°C in a humidified atmosphere containing 5% CO_2_ in 150 mm dishes (Life Technologies, Grand Island, NY). When the cultures reached near confluence (>80%), the cells were detached by treatment with trypsin/EDTA and replated at a density of 4000cells/cm^2^. The ASCs were passaged up to a maximum of four times. These cells have been characterized in our previous publication [20]. Methods for culture of educated macrophages were modified from the techniques published by Kim and Hematti [15]. Macrophages (2 × 10^6^ cells/well) were placed at the bottom of 6 well plates. Then, ASCs (5 × 10^5^ cells/well, a ratio of 4 : 1) were added on the top of Transwell (0.4 *μ*m pore size; Costar; Corning) system. After 48 hours of coculture, macrophages were harvested and designated as “educated macrophages” and used for in vivo experiment.

### 2.4. LPS-Induced Systemic Response

LPS from* Escherichia coli* O111:B4 (Sigma, St. Louis, MO) was dissolved with phosphate-buffered saline (PBS) by 1 mg/mL. Mice were randomly assigned to one of five experimental groups: (1) PBS control; (2) LPS; (3) LPS + ASCs; (4) LPS + untreated macrophages; or (5) LPS + educated macrophages. Mice were inoculated intraperitoneally with 5 mg/kg LPS or PBS. Before infusion, ASCs, educated macrophages, and untreated macrophages were washed with warm PBS and resuspended at a concentration of 1 × 10^6^ cells per 0.2 mL of PBS. Immediately after PBS or LPS treatment, 1 × 10^6^ cells (ASCs, educated macrophages, or untreated macrophages) or PBS (0.2 mL) were injected via the tail veins of the mice. Animals were weighed and sacrificed before and at 6, 24, and 48 h after LPS or PBS treatment. Lungs were harvested for histological analysis and determination of the wet-dry ratio. Blood and bronchial alveolar lavage (BAL) samples were collected from animals for cytokine analysis.

### 2.5. Wet-Dry Analysis

Lungs were removed from mice, placed into microcentrifuge tubes, and weighed. Lungs were then desiccated at 85°C oven for 48 hours for dry weight measurement. The wet-dry ratio was determined by dividing the weight before and after desiccating.

### 2.6. Histopathology

To harvest the lungs, the tracheas were cannulated and the lungs fixed by inflation with 4% paraformaldehyde. Following overnight fixation, tissue was embedded in paraffin and sectioned at 5 *μ*m thickness. Hematoxylin and eosin (H&E) staining was performed to determine morphology and inflammatory infiltrate. For measurement of the number of neutrophils in the lungs, modifications were made from the technique published by Everhart et al. [21]. Briefly, numbers of neutrophils were counted at 10 randomly selected high-power magnification fields (×400) in three histological sections per mouse from a total of four mice.

### 2.7. Detection of Cytokines

BAL samples were collected using a standard protocol. Mouse blood samples were collected and allowed to clot at room temperature for 30–90 min, followed by centrifugation at 400 g for 6 min. The serum and supernatants from BAL samples were then aliquoted and stored at −80°C. All the samples were analyzed for IL-1*β*, IL-4, IL-6, IL-10, IL-13, IL-17A, IFN-*γ*, TNF-*α*, and MIP-1*α* via a ProcartaPlex*™* Multiplex Immunoassay (eBioscience, San Diego, CA) according to the manufacturer's protocol. The assay plate was read on the Luminex 200 System (Luminex, Austin, TX). Standards and samples were run in duplicate and analyzed using the ProcartaPlex Analyst Software 1.0.

### 2.8. Coculture Experiments

Mouse peritoneal macrophages (2 × 10^6^) were cultured alone or cocultured with ASCs (5 × 10^5^) in either a standard 6-well or in a Transwell (0.4 *μ*m pore size; Costar; Corning) system for 48 hours. Macrophages were then stimulated with or without LPS (10 ng/mL) for 24 hours. The cell culture supernatants were harvested and stored at −80°C for future cytokine analysis. Macrophages were treated with trypsin and harvested for phenotype analysis. For the ARDS serum experiment, macrophages were incubated for 24 h with fresh culture medium plus 8% FBS (M group), normal human serum, or serum from ARDS patients. Then, macrophages were collected for phenotype analysis. For the IL-10 blockage experiment, macrophages were indirectly cocultured with ASCs via Transwell system in the presence or absence of IL-10 antibody (10 ng/mL) (mouse monoclonal antibody, R&D Systems, MAB417), IL-10 receptor antibody (10 ng/mL) (mouse polyclonal antibody, R&D Systems, AF-474-NA), or mouse IgG1 control (10 ng/mL) (R&D Systems). After 48 h, cells were replenished with fresh medium and treated with LPS (10 *μ*g/mL), and treatment was continued with IL-10 antibody, IL-10 receptor antibody, or IgG1 control for 24 h. Culture supernatants were harvested for cytokine assay. For the IL-10 treatment experiment, macrophages (2 × 10^6^ cells/well) were pretreated with or without IL-10 (1 ng/mL and 10 ng/mL) for 6 hours and then incubated with LPS (10 ng/mL) for another 24 hours. Culture supernatants and macrophages were harvested for mechanistic analysis.

### 2.9. Flow Cytometry

For determination of M2 macrophage expression, cells were labeled with FITC anti-mouse CD68 (BioLegend, San Diego, CA) and APC anti-mouse CD206 (BioLegend). After incubation with antibodies at 4°C for 30 min, cells were washed with PBS supplemented with 2% BSA, 4 mM EDTA, and 0.01% NaN3 and fixed in 4% paraformaldehyde. Data were collected on a FACSCalibur (BD Biosciences) and analyzed using FlowJo software.

### 2.10. Statistical Methods

Data are expressed as mean ± standard error of the mean (SEM). Statistical analysis was carried out using the GraphPad Prism software. Comparisons were analyzed by one-way ANOVA with a Bonferroni post hoc test or Student's* t*-test. Values were considered significant if *p* < 0.05.

## 3. Results

### 3.1. Educated Macrophages Mimic the Effect of ASCs in LPS-Induced Lung Inflammation

**Figure 1:**
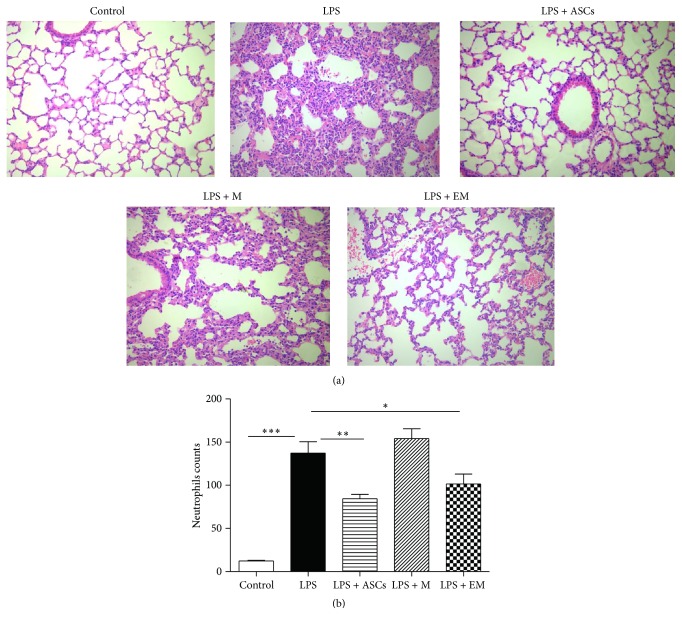
Educated macrophages (EM) decrease LPS-induced lung inflammation. Mice were allocated and received the following treatment: control, LPS, LPS + ASCs, LPS + macrophages (M), and LPS + EM. Animals were sacrificed before and at 6, 24, and 48 h after LPS or PBS treatment. (a) Lungs were fixed in paraformaldehyde. Lung sections were stained with H&E and visualized at ×200 magnification. (b) Quantification of neutrophils per high-power field on lung sections stained with H&E. All data are expressed as mean ± SEM. ^*∗*^ *p* < 0.05; ^*∗∗*^ *p* < 0.01; and ^*∗∗∗*^ *p* < 0.001.

To determine whether administration of educated macrophages alters LPS-induced lung inflammation, mice were randomized to the following 5 groups: control, LPS, LPS + ASCs, LPS + untreated macrophages (M), and LPS + educated macrophages (EM). At the 24 h time point, histological sections stained with H&E are shown in Figure 1(a). Administration of LPS revealed evidences of lung inflammation including marked inflammatory infiltrates, alveolar septal thickening, and interstitial edema. Infusion of ASCs or educated macrophages reduced airspace inflammation. Infusion of untreated macrophages did not inhibit the effect of LPS in the lung. To quantify the effect of educated macrophages on lung inflammation, a blinded histopathological examination of lung sections was carried out and the neutrophils in histological sections were counted at the 24 h time point (Figure 1(b)). Significant decreases in the number of neutrophils in animals treated with ASCs or educated macrophages were revealed as compared with LPS alone.

**Figure 2:**
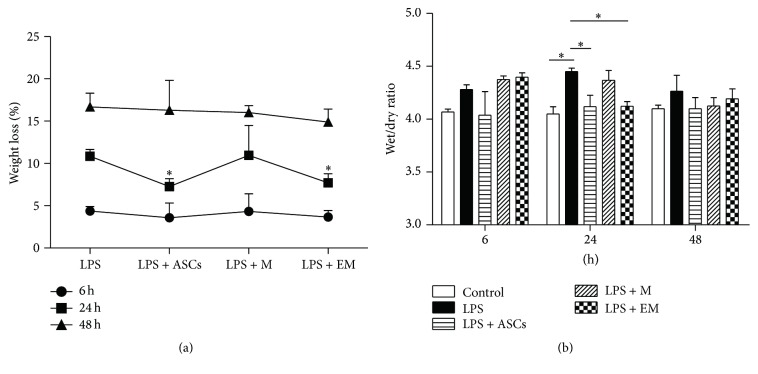
Infusion of educated macrophages (EM) reduces weight loss and pulmonary edema in mice treated with LPS. Animals were sacrificed before and at 6, 24, and 48 h after LPS or PBS treatment. (a) Percentage of weight loss was determined by the lost weight divided by the weight before LPS treatment. (b) Pulmonary edema was measured as the wet-dry ratio. M = macrophages. All data are expressed as mean ± SEM; *n* = 4 per group. ^*∗*^ *p* < 0.05.

ASCs have been shown to alleviate the weight loss of LPS-injured mice [22]. Educated macrophages had a similar effect to that of ASCs in reducing weight loss at 24 h time point (Figure 2(a), *p* < 0.05). Pulmonary edema is a hallmark of lung edema and inflammation.  Figure 2(b) summarized the time course of wet-dry weight ratios of lungs from all 5 groups. In mice treated with LPS alone, edema reached a peak at 24 h and was largely resolved by 48 h, which is similar to the animals that received LPS + M. No edema developed in the lungs of animals received LPS + ASCs and LPS + EM. These findings demonstrate that educated macrophages reduce LPS-induced lung inflammation.

### 3.2. Educated Macrophages Alleviate the LPS-Induced Systemic Inflammatory Response

**Figure 3:**
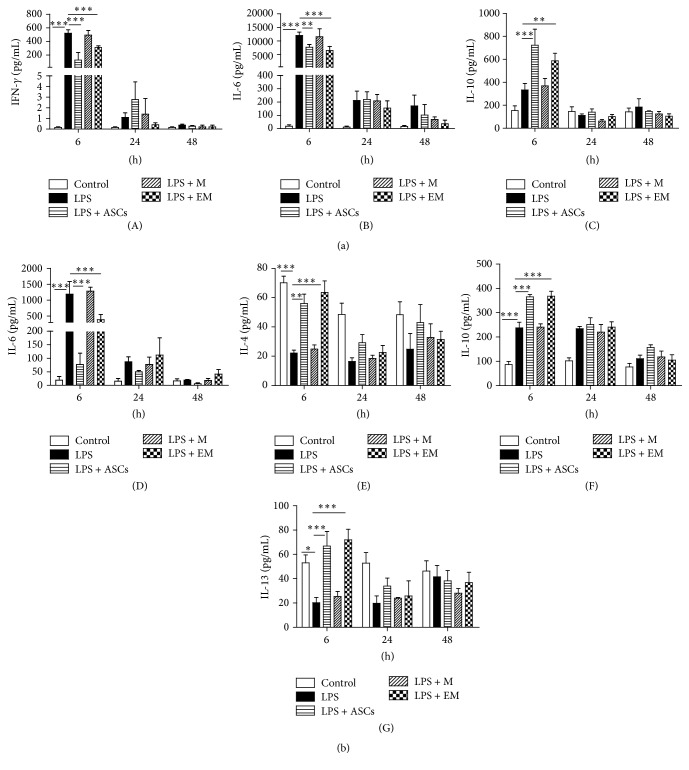
Infusion of educated macrophages (EM) alters the systemic inflammatory response to LPS. In each group, blood and BAL samples were collected at 6, 24, and 48 h after LPS or PBS treatment. Cytokine concentrations were determined in a Luminex system. (a) Cytokine levels in serum (IFN-*γ*, IL-6, and IL-10). (b) Cytokine levels in BAL (IL-4, IL-6, IL-10, and IL-13). M = macrophages. All data are expressed as mean ± SEM; *n* = 4 per group. ^*∗*^ *p* < 0.05; ^*∗∗*^ *p* < 0.01; and ^*∗∗∗*^ *p* < 0.001.

To evaluate the anti-inflammatory actions by ASCs and educated macrophages, levels of proinflammatory and anti-inflammatory cytokines were measured in serum and BAL collected from the above animals. Six hours after LPS administration, treatment with ASCs and educated macrophages significantly attenuated the levels of LPS-induced proinflammatory cytokine IFN-*γ* and IL-6 while increasing the level of anti-inflammatory cytokine IL-10 in the serum (Figure 3(a)). ASCs and educated macrophages also significantly decreased IL-6 level and elevated levels of IL-4, IL-10, and IL-13 in the BAL (Figure 3(b)). This response peaked at 6 h after LPS administration and largely subsided by 48 h. The effect of ASCs observed in the present study correlates well with the literature [8, 22]. These results demonstrate that educated macrophages reduce LPS-induced systemic cytokine response.

### 3.3. ASCs Elevate Expression of M2 Macrophages Independent of Direct Cell-to-Cell Contact

**Figure 4:**
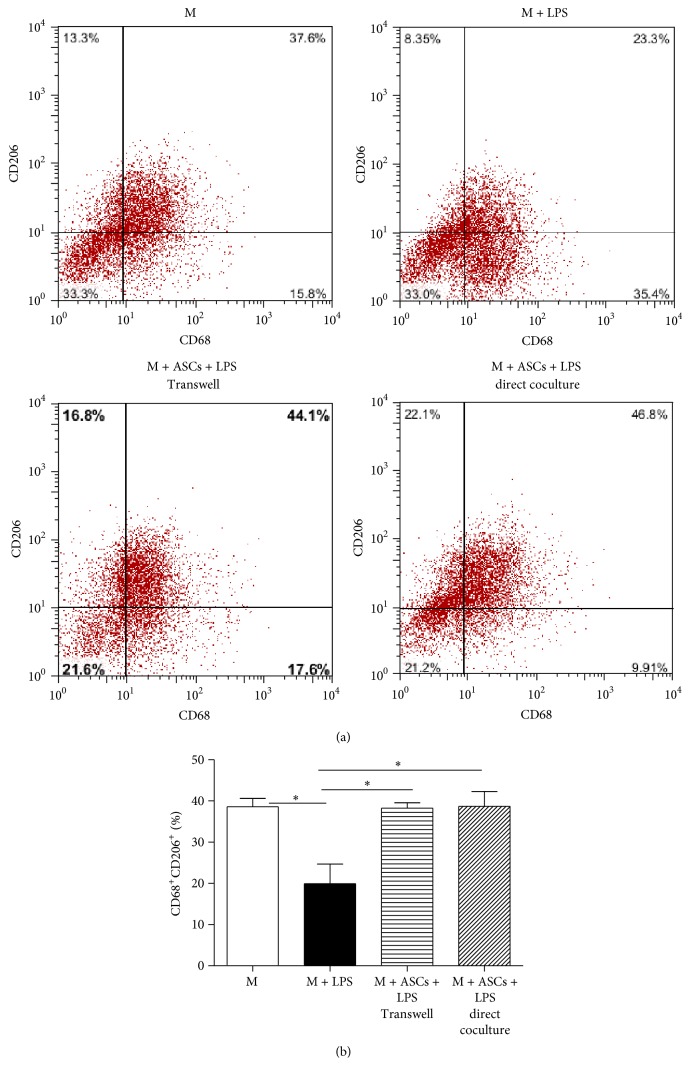
ASCs increase the expression of CD206^+^CD68^+^ macrophages independent of direct cell-to-cell contact. (a) Macrophages (M) (2 × 10^6^ cells/well) were cultured alone or cocultured with ASCs for 48 h via Transwell or direct culture (M : ASCs ratio = 4 : 1). Cells were then washed and incubated with fresh culture medium for 24 h with or without LPS (10 ng/mL). The expression of CD206^+^CD68^+^ M was analyzed via flow cytometry. (b) Percentage of CD206^+^CD68^+^ macrophages. All data are expressed as mean ± SEM; *n* = 4 per group. ^*∗*^ *p* < 0.05.

To determine the mechanisms of ASCs in alleviating systemic inflammatory response, we studied cell surface expression of CD68^+^CD206^+^, a well known marker for M2 macrophages [23]. Macrophages were cultured alone, cocultured for 48 h with ASCs via Transwell or direct coculture and incubated with or without LPS (10 ng/mL) for an additional 24 h. LPS treatment significantly decreased the levels of CD68^+^CD206^+^ cells when macrophages were cultured alone (Figures 4(a) and 4(b)). Both Transwell and direct culture with ASCs reversed the effect of LPS on CD68^+^CD206^+^ cells. The data suggest that paracrine factors are involved in the increased M2 expression.

### 3.4. ASCs Increase M2 Expression in Macrophages Treated with ARDS Serum

**Figure 5:**
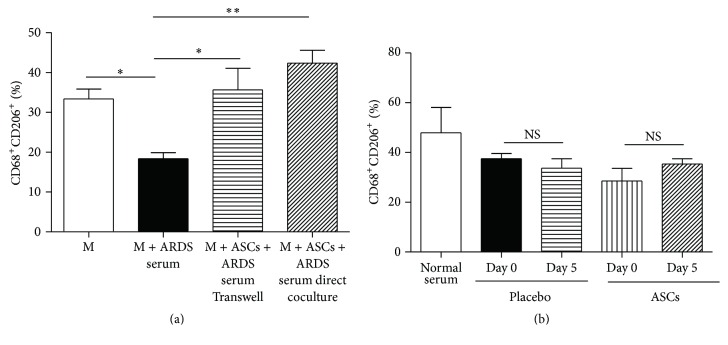
ASCs increase the levels of CD68^+^CD206^+^ macrophages treated with ARDS serum. (a) Peritoneal macrophages (M) (2 × 10^6^ cells/well) were cultured alone, cocultured directly with ASCs, or cocultured with ASCs via Transwell (M : ASCs ratio = 4 : 1) for 48 h. Cells were then washed and incubated for 24 h with fresh culture medium plus 8% FBS (M group) or 8% pooled serum from ARDS patients collected at the peak of their clinical symptoms. *n* = 4 per group. (b) Serum samples for culture medium (8%) were from healthy subjects or ARDS patients treated with placebo or ASCs in a previous clinical trial. *n* = 3 per group. The expression of CD206^+^CD68^+^ cells was analyzed via flow cytometry. NS = nonsignificant. All data are expressed as mean ± SEM. ^*∗*^ *p* < 0.05 and ^*∗∗*^ *p* < 0.01.

To investigate the mechanism of ASCs for the potential treatment of ARDS, macrophages were cultured with medium containing 8% fetal bovine serum or pooled ARDS serum collected at the peak of clinical symptoms. Similar to LPS, ARDS serum significantly reduced the expression of CD68^+^CD206^+^ (M2). Both direct coculture and Transwell of ASCs with macrophages restored the expression of M2 macrophages (Figure 5(a)). In a clinical trial for ARDS conducted by our group, SP-D levels, a marker for ARDS, were significantly reduced after ASCs treatment although no clinical beneficial effect was noticed [20]. The serum samples from patients treated with placebo and ASCs in the trial were cultured with macrophages and examined for M2 expression. There was no significant difference in M2 expression before and after treatment in both placebo and ASCs groups (Figure 5(b)).

### 3.5. Alterations in the Cytokine Profile Produced by Educated Macrophages Are Mimicked by IL-10 Treatment

**Figure 6:**
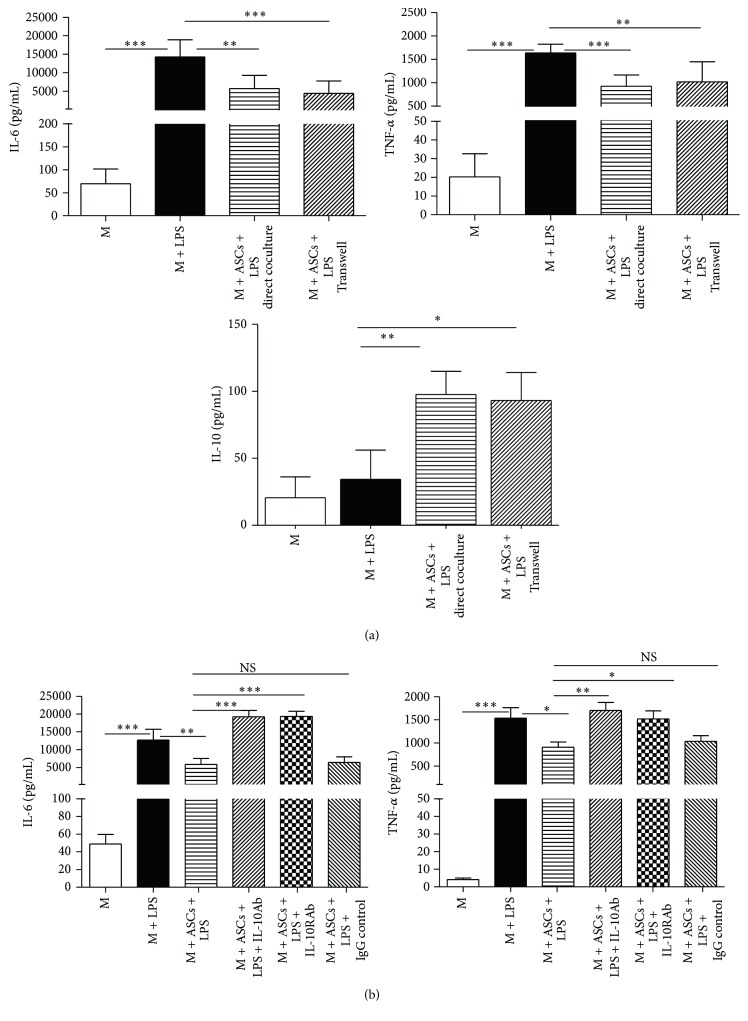
ASCs and IL-10 regulate the profile of cytokines produced by macrophages (M). (a) M (2 × 10^6^ cells/well) were cultured alone, cocultured directly with ASCs, or cocultured with ASCs via Transwell (M : ASCs ratio = 4 : 1) for 48 h. Cells were then washed and incubated for 24 h with fresh culture medium with or without LPS (10 ng/mL). (b) Neutralizing IL-10 antibody (IL-10Ab, 10 ng/mL), IL-10 receptor antibody (IL-10RAb, 10 ng/mL), or IgG control (10 ng/mL) was added to the medium at the beginning of the Transwell cocultures of ASCs + M. Then, cells were treated with or without LPS (10 *μ*g/mL) for another 24 h. Culture supernatants were harvested for cytokine assay via Luminex. All data are expressed as mean ± SEM; *n* = 4 per group. ^*∗*^ *p* < 0.05; ^*∗∗*^ *p* < 0.01; and ^*∗∗∗*^ *p* < 0.001.

To further confirm that ASCs promote the anti-inflammatory M2, the secretion of cytokines in cultured macrophages was examined. Macrophages were cultured for 2 days alone, via direct coculture with ASCs or Transwell. Then, cells were treated with or without LPS (10 ng/mL) for an additional 24 h and cytokine levels in the supernatants were determined. The levels of proinflammatory IL-6 and TNF-*α* were significantly elevated following LPS treatment and were significantly decreased by direct coculture or Transwell with ASCs (Figure 6(a)). In contrast, the levels of anti-inflammatory IL-10 were significantly augmented with both ways of coculture (Figure 6(a)). Similar to the previous findings in the expression of M2 macrophages (Figure 4), there was no statistical difference in cytokine levels (IL-6, TNF-*α*, and IL-10) between direct coculture and Transwell.

IL-10 has been shown to mediate the response of mesenchymal stem cells on macrophages in sepsis [10]. To determine whether IL-10 mediates the alteration of inflammatory cytokines of ASCs, macrophages were cocultured with ASCs via Transwell system in the presence of neutralizing IL-10 antibody (10 ng/mL) or IL-10 receptor antibody (10 ng/mL). Cytokine levels in the culture supernatants were analyzed after treatment with LPS for 24 h. Both antibodies for IL-10 and IL-10 receptor significantly impaired the ability of ASCs to inhibit the production of proinflammatory cytokines IL-6 and TNF-*α* by macrophages (Figure 6(b)).

**Figure 7:**
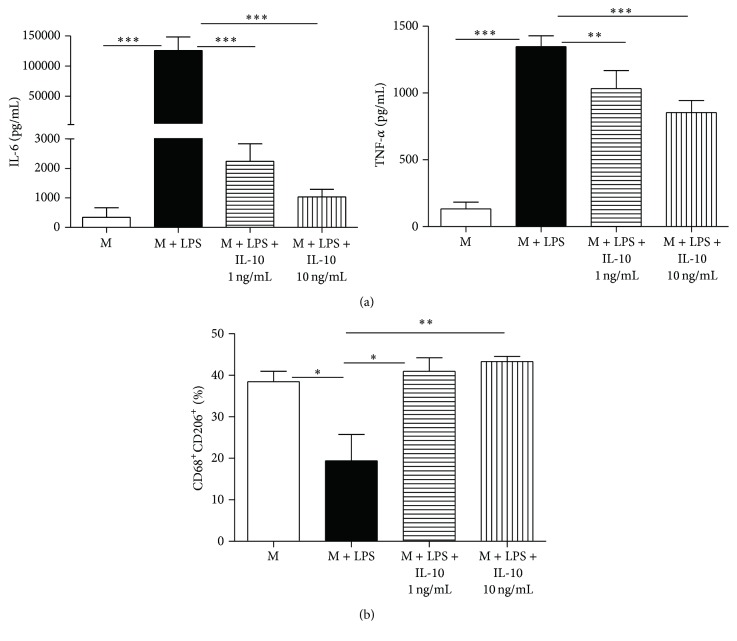
IL-10 mimics the effects of educated macrophages. (a) M (2 × 10^6^ cells/well) were pretreated with or without IL-10 (1 ng/mL and 10 ng/mL) for 6 hours and then incubated with LPS (10 ng/mL) for another 24 hours. Culture supernatants were harvested for cytokine assay via Luminex. (b) Percentage of CD68^+^CD206^+^ macrophages after IL-10 and LPS treatment was determined via flow cytometry. All data are expressed as mean ± SEM; *n* = 4 per group. ^*∗*^ *p* < 0.05; ^*∗∗*^ *p* < 0.01; and ^*∗∗∗*^ *p* < 0.001.

To further confirm the roles of IL-10 in the educated macrophages, macrophages were cultured with or without IL-10 (1 ng/mL and 10 ng/mL) for 24 hours. M2 expression and cytokine levels in the culture supernatants were determined. Unsurprisingly, IL-10 reduced proinflammatory cytokine IL-6 and TNF-*α* expression (Figure 7(a)). IL-10 also induced M2 phenotype (Figure 7(b)). These findings suggest that ASCs regulate cytokine expression and phenotype in macrophages via an IL-10-dependent mechanism.

## 4. Discussion

In the present study, we tested a new cell-based therapeutic strategy for systemic inflammation using macrophages educated via coculture with ASCs. Our results revealed that educated macrophages directly ameliorated lung inflammation and reduced weight loss as well as pulmonary edema in mice treated with LPS. Educated macrophages also decreased the proinflammatory cytokine levels and increased the anti-inflammatory cytokine levels in serum and BAL of the animals. In vitro, ASCs elevated expression of M2 macrophages independent of direct cell-to-cell contact when macrophages were treated with LPS or ARDS serum. The polarization of M2 macrophages in educated macrophages is characterized by a reduced ability to produce proinflammatory cytokines IL-6 and TNF-*α* and an increase in IL-10 level. In addition, the effects of ASCs on macrophages were blocked by IL-10/IL-10 receptor antibody and mimicked by IL-10.

The direct therapeutic effect of educated macrophages has been documented in two prior studies. In a mouse model of acute kidney injury, animals treated with M0 and M1 macrophages suffered a more severe histological and functional injury, while animals infused with educated macrophages showed mild manifestation [18]. In another report, macrophages were educated via culture with conditioned medium from ASCs [17]. Systemic infusion of the educated macrophages inhibited colitis in mice and reduced mortality as well as weight loss while lowering the colonic and systemic levels of inflammatory cytokines. Importantly, therapeutic injection of the macrophages in established chronic colitis alleviated disease progression and avoided recurrence. Moreover, the macrophages protected cecal ligation and puncture-induced sepsis [17].

The concept of educated macrophages was first created by Kim and Hematti. They found that macrophages adopted the phenotypes for M2 after 3 days of coculture with BM-ASCs [15]. González et al. cocultured colitis-derived macrophages with ASCs and found a decrease in the proinflammatory cytokines TNF-*α* and IL-12 along with an elevation in the anti-inflammatory IL-10. Furthermore, the effects of ASCs on cytokines were reversed by PGE2 blockage [24]. Another group found that BM-MSCs markedly affect the function of macrophages. Educated macrophages had a reduction of the inflammatory cytokines TNF-*α*, IL-6, IL-12p70, and IFN-*γ* and an increase of IL-10 and IL-12p40 when stimulated with LPS [25]. When cocultured with gingival ASCs, Zhang et al. discovered that macrophages acquired an anti-inflammatory M2 phenotype characterized by an increased expression of CD206 and IL-10 as well as a suppressed production of tumor TNF-*α*. In vivo, gingival ASCs homed to the wound site, promoted M2 polarization, and significantly enhanced wound repair. The gingival ASCs-induced suppression of TNF-*α* secretion by macrophages appears to correlate with impaired activation of NF*κ*B p50 [26].

In the present study, we determined that ASCs were able to elevate expression of M2 macrophages and modified cytokine expression without direct cell-to-cell contact. The data suggest that ASCs modulate the function of macrophages via soluble factors or newly defined extracellular vesicles [12]. Our findings were supported by reports from other studies. In an animal model of postspinal cord injury, encapsulated human ASCs were able to modulate the function of inflammatory macrophages and promoted the alternative M2 macrophage phenotype [27]. In vitro, this was evidenced by a reduction in macrophage iNOS expression with a concomitant increase in CD206 [27]. In another study, macrophages cultured with ASCs-conditioned medium showed a M2 phenotype which is characterized by high arginase activity, increased production of IL-10 upon restimulation, and potent immunosuppressive activity on T cells and macrophages [17]. Although our data showed that both ASCs and educated macrophages were able to reduce LPS-induced systemic response, ASCs might have a better regulatory effect as documented in data from neutrophil infiltration (Figure 1) and cytokine levels (Figure 3). In addition to educated macrophages, ASCs may be able to act via other mechanisms. For example, a number of studies have shown the capacity of mesenchymal stem cells to promote the generation of regulatory T cells (Treg) by activating the Notch 1 signaling pathway [28] or through production of HLA-G5 [29]. Treg itself possesses the ability to ameliorate the LPS response [30].

By depletion of circulating monocytes using clodronate, Németh et al. have demonstrated that BM-MSCs attenuate sepsis via prostaglandin E2-dependent reprogramming of host macrophages to increase their IL-10 production. They propose that MSCs are activated by LPS or TNF-*α*. Then, MSCs reprogram macrophages by releasing prostaglandin E2 that acts on the macrophages through the prostaglandin receptors. Next, activated macrophages produce anti-inflammatory IL-10 which reduce inflammation [10]. M2 macrophage activation has been reported as one of the mechanisms of BM-MSCs in alleviating lung injury by Ionescu et al. [31]. MSCs-derived conditioned medium (MSCs-CdM) promoted the resolution of LPS-induced lung injury by increasing a wound healing/anti-inflammatory M2 macrophage phenotype. MSCs-CdM increased arginase-1 activity and Ym1 expression in LPS-exposed alveolar macrophages. In vivo, alveolar macrophages from LPS-MSCs and LPS-MSCs-CdM lungs had enhanced expression of Ym1 and decreased expression of inducible nitric oxide synthase compared with untreated LPS mice. This suggests that MSCs-CdM promotes the alternative macrophage activation to an M2 “healer” phenotype. Furthermore, recombinant insulin-like growth factor I (IGF-I) partially reproduced the lung protective effect of MSCs-CdM [31]. By injecting ASCs-educated macrophages into mice treated with LPS, the present study showed that educated macrophages alone can directly ameliorate systemic inflammation. This finding may provide new insights into the mechanisms of MSCs and systemic inflammation.

The use of allogeneic MSCs holds great promise as a treatment for ARDS, sepsis, and systemic inflammation due to time limitations and the difficulties involved with harvesting autologous BM-MSCs or ASCs in these critical ill patients. Allogeneic BM-MSCs and ASCs have been efficiently used without major histocompatibility complex matching in clinical studies such as graft-versus-host disease [32], myocardial infarction [33], and inflammatory bowel diseases [34]. In a study of two ARDS patients administered with a dose of 2 × 10^6^ MSCs cells per kilogram, both patients improved with resolution of respiratory, hemodynamic, and multiorgan failure [35]. Our group previously conducted a pilot clinical study in the treatment of ARDS with 1 × 10^6^ ASCs cells per kilogram. The results showed that there were no short-term toxicities or serious adverse events related to ASCs administration. In the ASCs group, serum SP-D levels at day 5 were significantly lower than those at day 0 (*p* = 0.027) while the changes in IL-8 levels were not significant. The IL-6 levels at day 5 showed a trend towards lower levels as compared with day 0, but this trend was not statistically significant (*p* = 0.06). However, length of hospital stay, ventilator-free days, and ICU-free days at day 28 after treatment were similar between ASCs and control group [20]. In the present study, when macrophages were treated with serum from the trial, there was no difference in M2 macrophage levels before and after ASCs treatment. This finding further indicates that our trial protocol is not optimal. A higher or more frequent dose may be warranted.

Based on the findings of the present study, educated macrophages can be obtained by 48 h of coculture ASCs with macrophages, which can readily be generated from peripheral blood monocytes. Therefore, educated macrophages may serve as an attractive and alternative treatment option for ARDS, sepsis, and systemic inflammation. In comparison with BM-MSCs or ASCs, treatments based on educated macrophages are limited to their use only in an autologous manner. This suggests an obvious disadvantage versus treatments with allogeneic BM-MSCs or ASCs. However, the long-term safety of BM-MSCs and ASCs is unknown due to concern for carcinogenesis [36]. Moreover, autologous macrophages have been successfully used for promoting transplant tolerance [37] and the treatment of chronic wounds [38] in clinical trials.

Our results demonstrate that administration of ASCs-educated macrophages reduce systemic inflammation induced by LPS. The data also reveal that ASCs favor the expression of M2 phenotype and anti-inflammatory cytokines independent of direct cell-to-cell contact. Furthermore, educated macrophages may function via IL-10 pathways. Further studies are warranted to delineate the mechanisms of educated macrophages on systemic inflammation.
